# Surgery of Motor Eloquent Glioblastoma Guided by TMS-Informed Tractography: Driving Resection Completeness Towards Prolonged Survival

**DOI:** 10.3389/fonc.2022.874631

**Published:** 2022-05-27

**Authors:** Carolin Weiss Lucas, Andrea Maria Faymonville, Ricardo Loução, Catharina Schroeter, Charlotte Nettekoven, Ana-Maria Oros-Peusquens, Karl Josef Langen, N. Jon Shah, Gabriele Stoffels, Volker Neuschmelting, Tobias Blau, Hannah Neuschmelting, Martin Hellmich, Martin Kocher, Christian Grefkes, Roland Goldbrunner

**Affiliations:** ^1^ Department of General Neurosurgery, Center of Neurosurgery, Faculty of Medicine and University Hospital Cologne, University of Cologne, Cologne, Germany; ^2^ Department of Neurosurgery, University Hospital Mannheim, Mannheim, Germany; ^3^ Department of Stereotaxy and Functional Neurosurgery, Center of Neurosurgery, Faculty of Medicine and University Hospital Cologne, University of Cologne, Cologne, Germany; ^4^ Institute of Neuroscience and Medicine (INM‐4), Forschungszentrum Julich, Juelich, Germany; ^5^ JARA ‐ BRAIN ‐ Translational Medicine, Aachen, Germany; ^6^ Department of Neurology, RWTH Aachen University, Aachen, Germany; ^7^ Institute of Neuropathology, Faculty of Medicine and University Hospital Cologne, University of Cologne, Cologne, Germany; ^8^ Institute of Pathology and Neuropathology, University Hospital Essen, Essen, Germany; ^9^ Institute for Diagnostic and Interventional Radiology, Faculty of Medicine and University Hospital Cologne, University of Cologne, Cologne, Germany; ^10^ Institute for Medical Statistics and Computational Biology, Faculty of Medicine and University Hospital Cologne, University of Cologne, Cologne, Germany; ^11^ Department of Neurology, Faculty of Medicine and University Hospital Cologne, University of Cologne, Cologne, Germany

**Keywords:** high grade glioma, HGG, rolandic, CST, transcranial magnetic stimulation, functional outcome, diffusion tensor imaging, DTI

## Abstract

**Background:**

Surgical treatment of patients with glioblastoma affecting motor eloquent brain regions remains critically discussed given the risk–benefit dilemma of prolonging survival at the cost of motor-functional damage. Tractography informed by navigated transcranial magnetic stimulation (nTMS-informed tractography, TIT) provides a rather robust estimate of the individual location of the corticospinal tract (CST), a highly vulnerable structure with poor functional reorganisation potential. We hypothesised that by a more comprehensive, individualised surgical decision-making using TIT, tumours in close relationship to the CST can be resected with at least equal probability of gross total resection (GTR) than less eloquently located tumours without causing significantly more gross motor function harm. Moreover, we explored whether the completeness of TIT-aided resection translates to longer survival.

**Methods:**

A total of 61 patients (median age 63 years, m = 34) with primary glioblastoma neighbouring or involving the CST were operated on between 2010 and 2015. TIT was performed to inform surgical planning in 35 of the patients (group T; vs. 26 control patients). To achieve largely unconfounded group comparisons for each co-primary outcome (i.e., gross-motor functional worsening, GTR, survival), (i) uni- and multivariate regression analyses were performed to identify features of optimal outcome prediction; (ii), optimal propensity score matching (PSM) was applied to balance those features pairwise across groups, followed by (iii) pairwise group comparison.

**Results:**

Patients in group T featured a significantly higher lesion-CST overlap compared to controls (8.7 ± 10.7% vs. 3.8 ± 5.7%; p = 0.022). The frequency of gross motor worsening was higher in group T, albeit non-significant (n = 5/35 vs. n = 0/26; p = 0.108). PSM-based paired-sample comparison, controlling for the confounders of preoperative tumour volume and vicinity to the delicate vasculature of the insula, showed higher GTR rates in group T (77% vs. 69%; p = 0.025), particularly in patients with *a priori* intended GTR (87% vs. 78%; p = 0.003). This translates into a prolonged PFS in the same PSM subgroup (8.9 vs. 5.8 months; p = 0.03), with GTR representing the strongest predictor of PFS (p = 0.001) and OS (p = 0.0003) overall.

**Conclusion:**

The benefit of TIT-aided GTR appears to overcome the drawbacks of potentially elevated motor functional risk in motor eloquent tumour localisation, leading to prolonged survival of patients with primary glioblastoma close to the CST.

## Introduction

Despite the availability of advanced functional imaging techniques, the decision whether or not to resect high-grade gliomas in highly eloquent locations like the primary motor system remains a continued matter of debate. In addition to the risk of compromising health-related quality of life, the decision-making process is influenced by the concern that patients in whom motor function deteriorates after surgery may subsequently become ineligible for adjuvant tumour treatment, which could negate the beneficial effect of surgical tumour tissue reduction on survival (1, 2). In consequence, many patients with tumours closely adjacent to the corticospinal tract (CST) are treated by biopsy and definitive radio-chemotherapy, although there is substantial evidence that the complete removal of contrast-enhancing (CE-) tumour tissue (i.e., gross total tumour resection; GTR) improves the survival of glioblastoma patients (1).

Modern preoperative imaging techniques can inform the surgical decision-makers with valuable data regarding the individual location of functionally important structures at risk, e.g., the CST. The CST represents an essential component of the primary motor system with comparatively little potential of functional reorganisation to compensate for motor deficits in case of structural harm (3). Its individual localisation can be challenging in glioblastoma patients due to tumour mass effects. Tractography based on diffusion tensor imaging (DTI) can provide a robust estimate of individual CST localisation also in the neighbourhood of brain tumours, especially if informed by functional localisation techniques like neuronavigated transcranial magnetic stimulation (nTMS) (4).

The use of functional localisers like nTMS to define the primary motor cortex (M1) as the starting region of interest (ROI) for tractography is of particular importance in motor eloquent brain tumours since not only physiological variability (5) but also tumour-related functional reorganisation and anatomical shifts hamper its localisation based on pure brain atlas information (6, 7).

The pre- and intraoperative use of advanced functional localiser techniques such as nTMS and nTMS-informed tractography (TIT) can affect surgical decision-making (8). However, the question of whether the application of those techniques is associated with a more aggressive resection and might—as a consequence—influence survival, remains controversially debated (8, 9) and cannot be adequately addressed by comparison with historical cohorts due to substantial changes in the general surgical strategy and the availability of pre- and intraoperative aids.

In this cohort study, we investigated the effect of TIT-aided neuronavigation on gross-motor functional outcome and completeness of resection linked to survival in patients with motor eloquent glioblastoma. Results were compared to matched cases from the same period and institution. We hypothesised that the use of TIT allows for tumour resection in comparatively critical location (i.e., close to the CST) without a major increase in gross motor function risk. Moreover, we hypothesised that the preoperative planning based on TIT allows for realistic expectations regarding the intraoperative findings, leading to resection completeness (i.e., GTR rate) noninferior to a control group with comparatively less motor eloquent tumour location. Lastly, we set out to explore to which extent the surgical success translates to improved progression-free and overall survival (PFS and OS, respectively).

## Methods

### General Study Design

In this hospital-based cohort study of 61 patients with first diagnosed primary, motor eloquent glioblastoma, we, first, investigated differences in the characteristics of group T, receiving preoperative TIT for surgery planning upon request by the responsible neurosurgeon, compared to the control group C. As a consequence, we expected an overall more motor eloquent localisation of tumours in group T (i.e., higher overlap with the CST). Second, the influence of TIT-aided surgery planning and other potential factors on postoperative functional changes were explored (primary outcome: gross motor function; secondary outcome: Karnofsky Perfromance Status, KPS). Third, factors influencing GTR were analysed; propensity score matching (PSM) was applied to balance significant confounders across groups before comparing GTR outcome groups T and C (cf. section *Data Analysis*). Finally, uni- and multivariate Cox Proportional Hazards regression models were used to explore the influencing factors on survival (PFS, OS) in this specific cohort; PSM was applied to achieve a largely unbiased comparison of survival outcomes between patients with vs. without GTR.

### Patients

Between July 2010 and May 2015, a consecutive group of patients with primary glioblastoma was allocated to this single-centre study cohort, characterised by scheduled tumour resection (following the decision of an interdisciplinary tumour board) and tumour infiltration of the posterior frontal and/or the anterior parietal lobes, thus showing the proximity of the tumour margins to M1 and/or the CST. Exclusion criteria were poor general performance [KPS <50%; (10)] and contraindications for magnetic resonance imaging (MRI). Part of the patients received preoperative nTMS mapping of the motor cortex together with TIT of the corticospinal/corticobulbar tract as part of the preoperative functional diagnostics (TI**T** group T), whereas others did not receive tractography (**c**ontrol group C), based on the individual decision of the responsible neurosurgeon. All patients receiving nTMS and/or tractography in addition to established pre-/intraoperative aids were prospectively filed in an institutional registry, which was approved by the local ethics committee, and provided written informed consent. Of note, epilepsy was not considered a contraindication to single-pulse nTMS as long as effective anticonvulsant treatment was provided, consistent with current safety data (11). The decision of whether or not a patient with suspected glioblastoma neighbouring M1/the CST would receive TIT was made by the responsible team of neurosurgeons on an individual risk estimation basis. Of important note, during the allocation period, preoperative TIT was not yet regarded as an essential prerequisite of state-of-the-art neuro-oncological surgery in motor-eloquent location, and was not always actively requested by the responsible team of neurosurgeons; moreover, logistic reasons and the ineligibility or unwillingness of some patients to undergo TMS can be held responsible for the non-performance of TIT, altogether explaining why not all patients received preoperative TIT.

All patients underwent neuro-oncological surgery according to current standards, including surgery performed or directly supervised by a senior neurosurgeon specialised in neuro-oncological surgery, and the routine use of 5-aminolevulinic acid (5-ALA) for fluorescence-guided resection, intraoperative ultrasound, and usually intraoperative neuronavigation. Intraoperative neuromonitoring was performed according to the preference of the neurosurgeon, and generally consisted of direct cortical stimulation (DCS) using a monopolar probe. Only very sporadically, additional subcortical stimulation was performed (using the train-of-five technique). In patients of the T group, DCS was guided by the nTMS data, with functional hotspots serving as starting points for determination of the DCS intensities [cf. (12)]. Generally, the surgical strategy aimed at the complete removal of solid tumour tissue, characterised by bright 5-ALA fluorescence, and the additional removal of moderately/weakly fluorescent tissue in tumour regions regarded non-eloquent. Of note, an essential prerequisite for indicating tumour resection was the expected achievability of GTR without relevant functional harm. Exceptionally, large tumours with intracranial mass effect (associated with clinical symptoms and/or non-eligibility for radiooncological treatment) were also considered for surgery even if the functionally critical localisation of CE-tumour parts (e.g., involving the basal ganglia) led to the *a priori* intention of subtotal resection.

The histological diagnosis of all patients eligible for the study was retrospectively re-evaluated according to the World Health Organisation (WHO) classification of 2016 [cf. (13) for review] by a senior neuropathologist (co-author TB) based on the available information regarding the isocitrate dehydrogenase (IDH) 1/2 gene mutation status.

This study was conducted in accordance with the ethical standards of the Declaration of Helsinki (first adopted in 1969, last revised in 2013).

### nTMS Motor Mapping and nTMS-Informed Tractography

nTMS was conducted to map M1 using the eXimia NBS system (version 4.2, Nexstim Ltd., Helsinki, Finland) equipped with a figure-of-eight-shaped coil for biphasic magnetic stimulation and an integrated neuronavigation system using the individual T1-weighted (T1w) MRI sequence, as previously described (14). In brief: depending on the tumour location and clinical motor deficits, motor-evoked potentials (MEPs) were recorded from the abductor pollicis brevis muscle, the plantar toe flexor muscles and/or the anterior lateral third of the tongue using surface electrodes (Ambu Neuroline, Bad Nauheim, Germany). For mapping, the stimulation intensity was adjusted to 110% of the resting motor threshold (RMT) of the respective muscles. Starting at the centre (hotspot) of each body part representation, mapping was continued radially until no more nTMS-elicited MEPs (of at least 50 μV peak-to-peak amplitude) were found. After mapping, MEP data were manually cleared from data points with implausible latencies or unintended voluntary reinnervation [cf. (15)]. All surviving map data points and the corresponding map centres (hotspots) were exported as binary image files, and were made available for surgical planning (by integration in the neuronavigation software IplanNet^®^; Brainlab, Feldkirchen).

**Figure 1:**
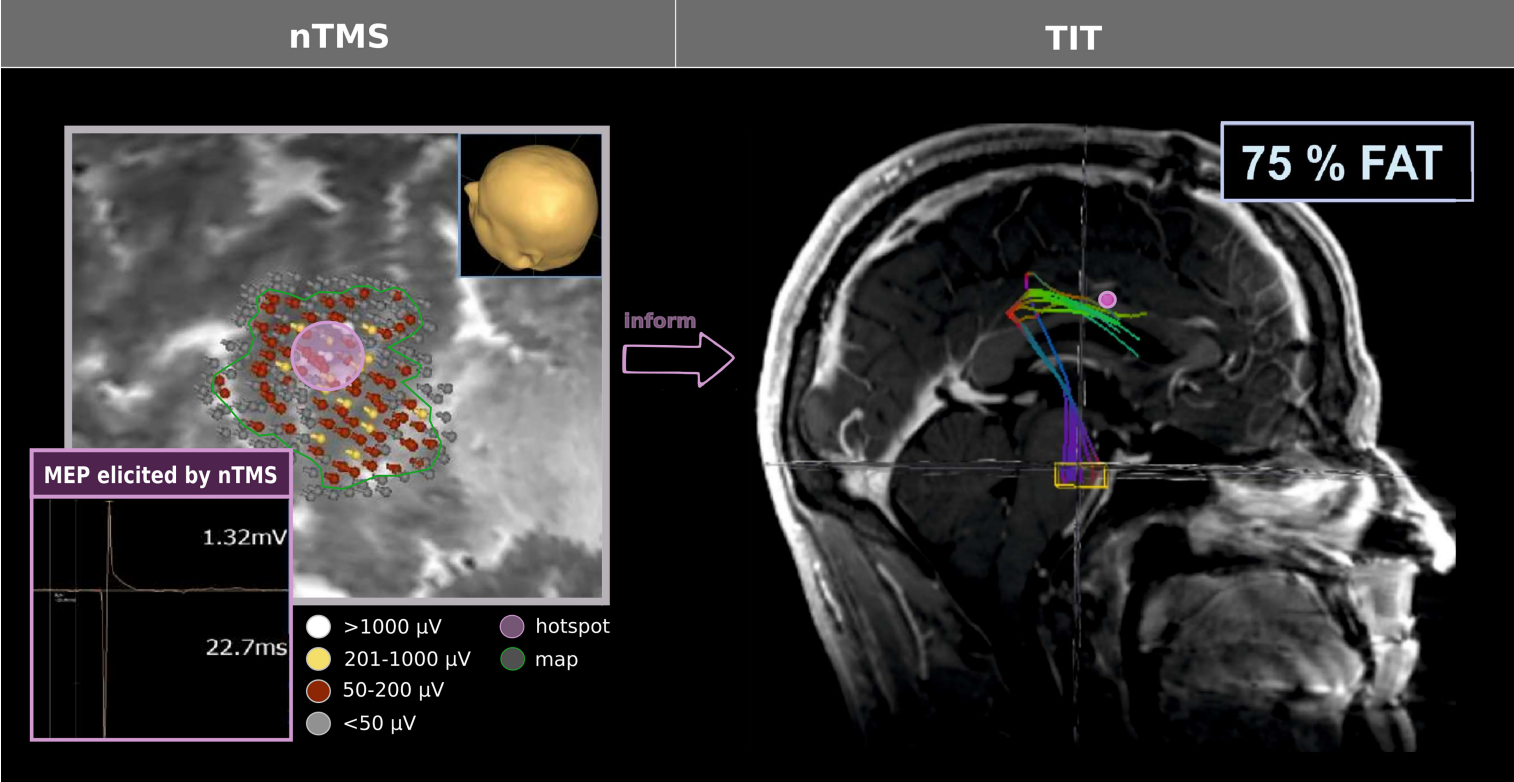
TIT workflow. nTMS was performed to achieve a functional map of the respective somatotopic area (here: hand representation). All map coordinates (corresponding to MEP of ≥50 μV peak-to-peak amplitude, i.e., labelled in red, yellow, or white) and the centre/hotspot coordinate (yielding the highest reproducible peak-to-peak amplitude; violet) were exported to inform tractography, serving as starting points for the deterministic algorithm. Notably, the centre/hotspot was enlarged by 5 mm; a second cubic region of interest was set in the anterior pontine region [yellow box; cf. Weiss Lucas et al. (15)]. Tractography was performed by adjusting the cut-off of fractional anisotropy values to the 75% of the fractional anisotropy threshold [FAT, according to Frey et al. (79)], exemplified here with the map centre/hotspot serving as the origin.

The somatotopic map centre volumes of interest corresponding to M1 were enlarged by a radius of 5 mm and served as origins for tractography of the somatotopic “core fibres” (terminating at a second region of interest in the anterior pontine region; **Figure 1**). Additionally, the total map extent was used as an alternative starting region of interest (to achieve a supposedly less specific but more sensitive tractography result). Tractography was performed by a deterministic algorithm using the software tool iPlanNet (Brainlab, Feldkirchen), based on the clinical DTI data (cf. MRI and Volumetric Analysis). Two patients with group T received tractography based on anatomical ROI seeding due to unavailability of nTMS data (including one case [ID 4] with a severe preoperative motor deficit impeding to elicit MEP by nTMS within the safety limits of stimulation intensity).

### MRI and Volumetric Analysis

All MRIs were obtained for clinical reasons. Preoperative MRI scans were acquired within ten days before surgery and included a T1w image sequence with and without gadolinium-based CE (post contrast), T2-weighted and fluid-attenuated inversion recovery (FLAIR) image sequences, and DTI meeting with current quality standards [i.e., ≥30 diffusion weighting directions (17)]. Postoperative scans were performed within 48 h after surgery, and comprised an additional DWI sequence, but no DTI.

The resection extent was determined based on the postoperative MRI. Here, any positive CE within brain tissue was assigned to tumour residual. GTR was considered the primary surgical outcome parameter due to its huge relevance for tumour control and survival of glioblastoma patients. Moreover, a quantitative, volumetric analysis tool (iPlan^®^, Brainlab, Feldkirchen, Germany) was used to measure the preoperative CE-tumour volume (PTV) and the residual CE-tumour volume (RTV) as follows (providing the volume in cm³): (i) based on CE-T1w MRI, all CE-tissue within the mask of the tumour region was labelled using auto-segmentation, (ii) vessels, bone and dural structures were excluded from the labelled volume, (iii) based on the fused non-CE-T1w MRI, all hyperintense structures included in the volume were removed. Thereafter, tumour volumes were exported and converted into NIfTI format for further postprocessing steps (cf. *Standardised Estimation of Tumour Eloquence*). Of note, in two cases images were unavailable in digital format (only as printed films); here, PTV was estimated based on two-dimensional measurements and the assumption of a spherical tumour model. GTR, PTV and RTV were independently assessed by two neurosurgeons (CS or AF, and CWL) and a certified neuroradiologist (HN) blinded to the group affiliation of the respective patient (T vs. C). Tumour mass reduction (i.e., the proportional extent of resection) was defined by ‘1-RRTV’, with the relative RTV (RRTV) representing the ratio between RTV and PTV.

### Standardised Estimation of Tumour Eloquence

**Figure 2:**
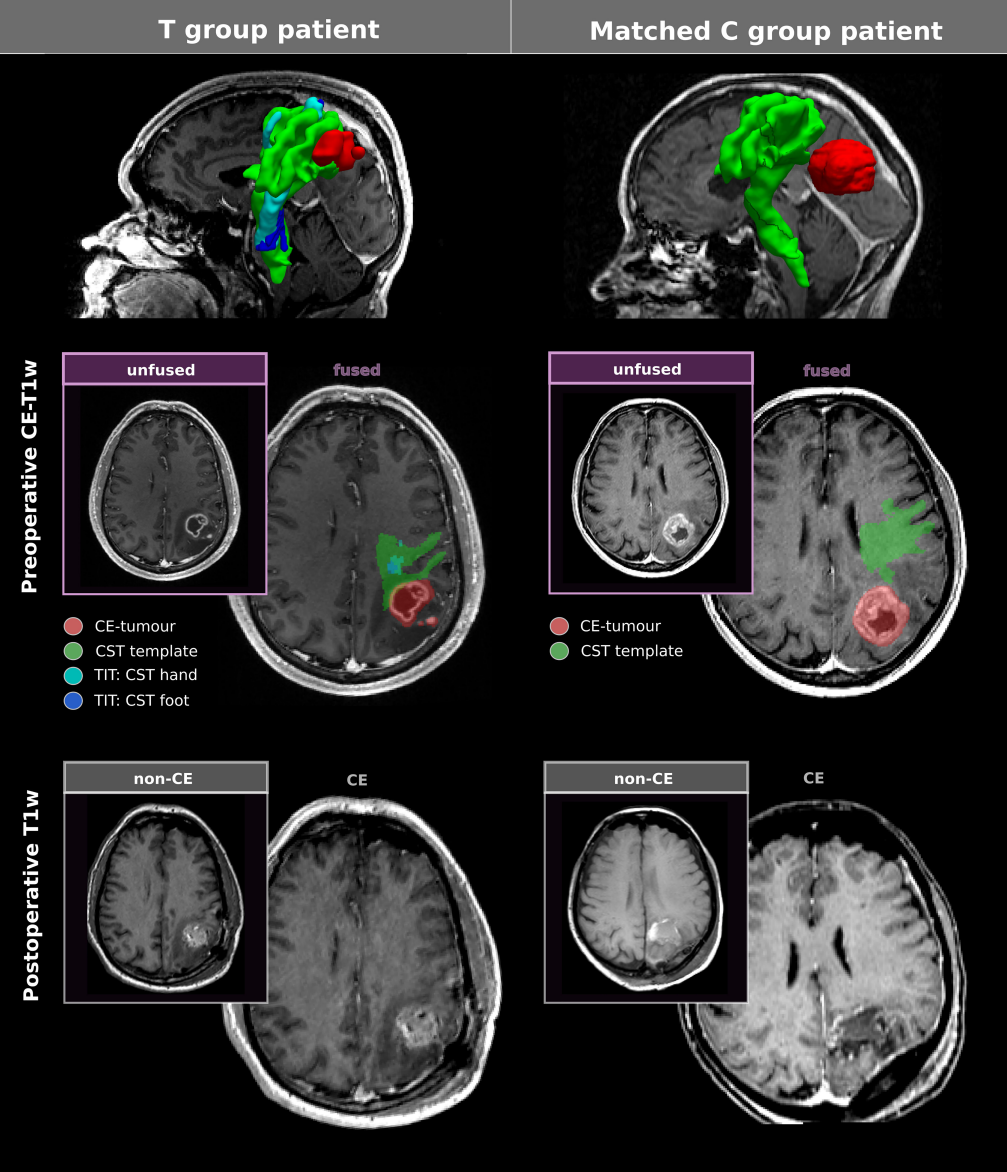
Illustration of clinical and experimental methodology. To investigate the influence of TIT on GTR, patients from group T (TIT) and control group C were pairwise matched according to the CE-tumour volume (PTV) and the insula-overlap. The figure illustrates the clinical and methodological imaging results in a PSM-paired couple of patients (IDs 35 and 48; **Supplementary Table S1**). In patients from group T (left), somatotopic CST fibres (cyan: hand; blue: foot) were reconstructed using TIT and were integrated in the neuronavigation system for surgery planning and intraoperative guidance. For a standardised assessment of tumour eloquence across groups, a probabilistic standard CST template (green) was used to compute the CST-overlap, i.e., the proportion of the CST template overlapped with the tumour volume (red). Case 35 (left): 50 year-old male patient presenting with focal motor seizures and coordination deficits responsive to oral antiepileptic and anti-oedematous drugs who underwent GTR aided by TIT, 5-ALA and DCS without postoperative gross motor deficits (PFS 7.3 months; OS 10.0 months). Case 48 (right): The 78 year-old female patient without preoperative motor deficits underwent GTR guided by neuronavigation and 5-ALA fluorescence without postoperative gross motor deficits (PFS 5.8 months; OS 8.6 months).

To provide a standardised estimate of the motor eloquence of the tumour location across all subjects, normalised CE-tumour volumes were overlaid with a population-based atlas template of the CST [(18) **Figure 2**]. Moreover, tumour overlap with the insula—representing a structure of high surgical risk due to the high likelihood of critical vascular damage—was assessed based on a standard atlas (19). To this end, tumour volumes were co-registered to Montreal Neuroimaging Institute (MNI) template space by linear and elastic registration of the underlying anatomical scans using the Advanced Normalisation Tools [ANTs (20)], which are comparatively robust to (tumour-induced) alterations of the cortical surface (21). This algorithm uses diffeomorphisms of both the input image (T1w of the patient) and the reference image (template T1w), commonly referred to as symmetric normalisation [SyN (20)], allowing for both small and large deformations. It is coupled with a difference metric, which evaluates the cross-correlation in neighbourhoods of voxels, and a multi-resolution optimisation. Visual inspection using well-known anatomical landmarks (e.g., corpus callosum, frontal horns of the lateral ventricles, central sulcus, outer brain margins) confirmed that normalisation was adequate. Proportional overlap volumes (i.e., overlap volume between tumour and the ipsilateral atlas template in relation to the respective atlas template volume) were calculated regarding the CST (CST-overlap) and the insular cortex (insula-overlap) by a custom-made Matlab script [using the “Tools for NIfTI and ANALYZE image” toolbox in MATLAB R2018b, The Mathworks, Massachusetts, IL, USA (22)].

The same script was applied to detect overlap (yes/no) between the tumour volume and other anatomical brain regions (frontal, parietal, temporal). A tumour extension to the temporal lobe was considered indicative of Sylvian fissure involvement—representing a second structure associated with increased risk of vascular damage –, and was manually checked on the original anatomical MRI (by CWL).

### Clinical Functional Assessment

To test the functional outcome, every patient underwent a neurological examination before surgery, on postoperative day 7 ± 3, and at follow-up after three months (i.e., 12 ± 4 weeks). Pre- and postoperative motor deficits were graded into four categories of muscle strength assessed from the most affected body part, based on the British Medical Research Council system for manual grading of muscle strength [MRC (23)], which was, however, developed for lesions of the peripheral nervous system: category 1 = no deficit (MRC 5/5); category 2 = slight deficit (MRC 4/5), category 3 = moderate deficit (MRC 3/5), and category 4 = severe deficit (MRC 0-2/5). To consider also the overall clinical condition, the KPS was assessed at each time point. Relative changes in gross motor function and KPS were described on a three-level ordinal scale (better, idem, worse). It is noteworthy that comorbidity (e.g., assessed by the Charlson Comorbidity Index) was excluded due to (i) lacking evidence regarding its impact on mortality and morbidity in patients with brain metastasis (24), and (ii) its relevance for glioma patients being critically debated (25).

### Survival

PFS and OS data were gathered by clinical follow-up documentation, or were obtained from the general practitioners of the patients, or from the designated mortality registry. The date of PFS was determined by the date of the MRI scan, which led to the statement of progressive disease (PD), or by the date of death, irrespective of its cause. PD statements were based on multidisciplinary consent in the weekly tumour board meeting. For the analysis of survival data, those long-term survivors, which were still alive at the time of manuscript preparation (n = 2), were censored. The time point of censoring was set to the date of the most recent patient contact.

### Data Analysis

The statistical analyses were performed using R (R Studio, Version 2021.09.2, based on R v4.1.2; libraries: psych, MASS, SURVIVAL, MatchIt, dplyr, optmatch, ggplot2).

The selection of features considered relevant in this study was based on evidence from previous studies, and was tailored to include all presumably independent factors, which were assessable before or at the time of surgery and had a potential influence on the chosen endpoints. Thus, the following factors were included:

*Functional outcome:* age (continuous), preoperative gross motor status (4 levels), PTV (continuous), CST-overlap (continuous), insula-overlap (continuous), involvement of the Sylvian fissure (dichotomous), tumour hemisphere (dichotomous), TIT (dichotomous).

*Resection outcome:* anti-oedematous drugs (dichotomous), PTV (continuous), CST-overlap (continuous), insula-overlap (continuous), involvement of the Sylvian fissure (dichotomous), tumour hemisphere (dichotomous), TIT (dichotomous).

*Survival outcome:* age (continuous), gender (dichotomous), promotor status of the O6-Methylguanin-DNS-Methyltransferase (MGMT; dichotomous), preoperative gross motor function (4 levels), preoperative KPS (11 levels), PTV (continuous), CST-overlap (continuous), insula-overlap (continuous), involvement of the Sylvian fissure (dichotomous), tumour hemisphere (dichotomous), TIT (dichotomous), GTR (dichotomous), RTV (continuous).

Data were analysed applying the following steps:

*(i) Regression analysis and feature selection:* Uni- followed by multivariate regression analyses were performed to select features (for survival: Cox proportional-Hazards regression using the “coxph” function from the {survival} library in R; for resection outcome: generalised linear logistic regression model using the “glm” function from the {MASS} library in R); here, a liberal statistical threshold of p ≤0.1 was applied to qualify factors for inclusion in the NULL model.

*(ii) Model simplification:* The NULL model was optimised stepwise by removal and addition of factors, based on the Akaike information criterion [AIC (26)] to compare the predictive value of models. Here, the automised “stepAIC” function (from the {MASS} library in R) was used. The increase in model complexity was only accepted if the improvement of the AIC was at least at the statistical level of p <0.1. The final model is referred to as the “winning model” throughout the manuscript. Whenever the winning model included factors other than the stratifying variable, PSM was performed in the following to control for confounders.

(iii) *PSM:* The concept of the PSM itself and of comparable methods have been implemented to improve data quality, comparability and estimation of treatment effects of groups by reduction or elimination of confounding effects [cf., e.g., (27)]. Therefore, this method has been widely regarded as the current gold standard for the analysis of observational data (28) and is of particular interest for the comparison of relatively small groups. Here, patients from the (larger) T group were matched pairwise according to the factors of the winning model to patients from the C group, based on their propensity scores. Here, the “matchit” function in R ({MatchIt} library, optimal matching method) was applied. The covariate balance in the matched sample was checked using paired-sample *post hoc* tests to exclude a significant group difference (with p <0.1 leading to rejection of the PSM result). This workflow (i–iii) was repeated for each distinct outcome (i.e., postoperative worsening of gross motor function, GTR, PFS, OS). Notably, TIT and GTR were stratifying factors for PFS/OS and thus not included in the PSM for these endpoints.

*(iv) Matched pair analysis:* Given optimal PSM regarding all variables of the winning model, tests for paired data (within-subject design) were applied for this part of the group comparisons (27): To test group differences between optimally matched dichotomous variables, McNemar’s chi-square test was used. Group comparisons regarding normally distributed (according to the Shapiro–Wilk test), continuous variables, were performed using two-sided t-tests. The Wilcoxon signed-rank test was applied for group comparison between rank scaled (e.g., KPS) and not normally distributed continuous paired data. Log-rank tests were applied for group comparison of survival models (using the “survdiff” function from the {survival} library in R).

A level of significance was set at p <0.05 (except for step [i] feature selection). For histograms, bin width was selected according to the rule of Freedman–Diaconis (29).

## Results

### Patients

A total of 61 patients (median age = 63 [32–87] years, 34 men) were included in the full data set (**Table 1**). Most patients (56% overall) presented with a preoperative gross motor deficit and received anti-oedematous medication (i.e., oral dexamethasone; 72% overall) before surgery; however, most patients were in good overall clinical status (median KPS = 90). Due to the inclusion criteria, patients were generally diagnosed with an IDH 1/2 wildtype glioblastoma, and 32% (n = 19) showed a promoter methylation of the MGMT gene (**Table 1**).

**Table 1:** Patient characteristics.

General	
Age (median in years *[range]*)	63 *[32;87]*
Gender	
N males (%)	34 (56%)
N females (%)	27 (44%)
Steroids preoperative (%)	44 (72%)
**Biological tumour characteristics**	
MGMT promoter methylated (%)	19 (32%)
IDH-1/2 status	
IDH1 R132H wildtype, immunohistochemical analysis (%)	55 (90%)
IDH1/IDH 2 wildtype, moleculargenetic analysis (%)	39 (63%)
Wildtype unknown (%)	5 (8%)
**CE-tumour location & size**	
Left hemisphere (%)	30 (49%)
Size (ccm, mean ± SD)	24 ± 20
Frontal^§^ (%)	47 (77%)
Parietal^§^ (%)	46 (75%)
Temporal^§^ (%)	15 (25%)
Insula^§^ (%)	20 (33%)
Sylvian fissure (%)	7 (12%)
*Elevated vascular risk (i.e., insula and/or Sylvian fissure)*	*22 (36%)*
CST involvement^§^ (%)	49 (82%)
Insula-overlap in mean ‰ ± SD	0.35‰ ± 0.11‰
CST-overlap in mean % ± SD	6.7% ± 9.2%
**Gross-motor function and general clinical condition**	
Any preoperative gross motor deficit (%)	34 (56%)
slight gross motor deficit (MRC 4/5)	19 (31%)
moderate gross motor deficit (MRC 3/5)	11 (18%)
severe gross motor deficit (MRC 0–2/5)	3 (5%)
KPS preoperative (median *[range; 0–100]*)	90 *[50;100]*
**Functional and tumour mapping/imaging**	
Preoperative	
NTMS	61%
DTI	57%
Intraoperative	
5-ALA	97%
Neuronavigation	93%
Intraoperative neuromonitoring (DCS)	64%

Pre- and intraoperative characteristics of the full patient cohort (n = 61) are provided. ^§^According to overlap between tumour volume and atlas template (yes/no). SD, standard deviation.

According to the tumour vicinity to the motor cortex and/or pyramidal tract, all tumours were characterised by frontal and/or parietal localisation, and partly involved the temporal lobe (25%), and/or reached the insula (33%), and/or the Sylvian fissure (12%). Notably, tumour infiltration reaching the insula and/or the Sylvian fissure (36% overall) was considered at increased risk of vascular damage (**Table 1**). Probable involvement of the CST and/or M1 was confirmed by the overlap of the normalised PTV with a standard CST map in 49 (82%) of the cases.

Surgery was almost always supported by intraoperative neuronavigation (93%) and 5-ALA fluorescence imaging (97%). The two remaining cases without 5-ALA administration (due to unexpected glioblastoma diagnosis; both T group) were not considered for PSM to avoid bias. Approximately 64% of the patients received intraoperative neuromonitoring, which generally consisted of DCS (except for two cases for which phase reversal was used to identify the central sulcus, and another two cases with additional use of subcortical stimulation). More than half of the patients were investigated by nTMS motor mapping (61%) and/or DTI-tractography (57%; group T) for surgery planning (**Table 1**). Notably, the size of the control group C was comparatively small (n = 26; 43%) since, in a given time frame, most patients with posterior frontal and/or anterior parietal tumour location were scheduled for TIT.

**Figure 3:**
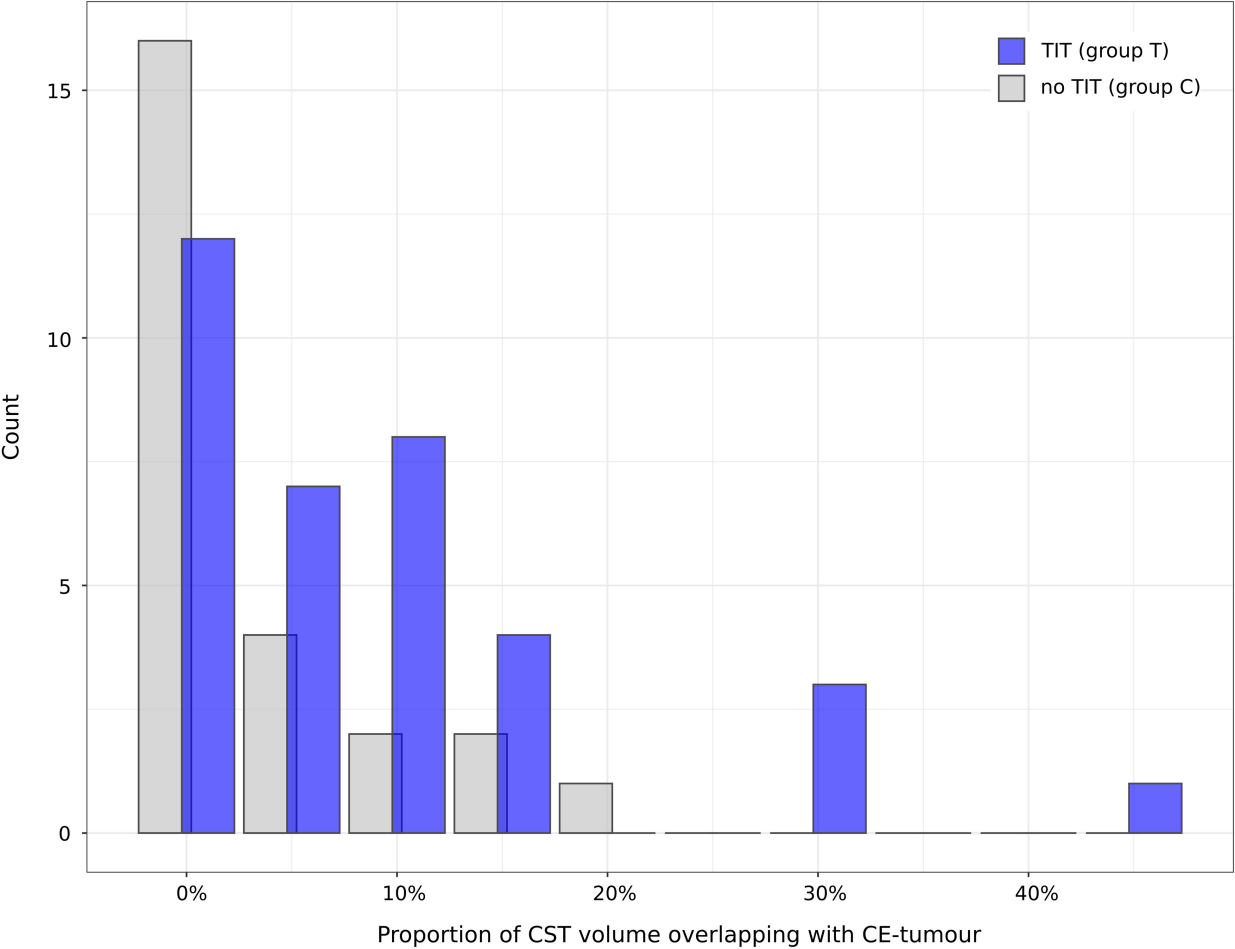
Groupwise distribution of CST-overlap. The histogram displays the distribution of the proportional CST volume overlapping with CE-tumour (CST-overlap), grouped by TIT. The Wilcoxon test revealed a higher mean CST-overlap in group T compared to group C (8.7% ± 10.7% vs. 3.8% ± 5.7%; p = 0.022). X-axis: percentage of CST-overlap. Y-axis: count. Blue: group T (TIT; n = 35). Grey: group C (control, no TIT; n = 26).

A comparison of means revealed no significant differences between cohort T (n = 35; median age = 60 [32–80] years, 18 men) and cohort C (n = 26; median age = 68 [51–87] years, 16 men) regarding the patient/tumour characteristics except for younger age (p = 0.046) and higher CST-overlap in group T (8.7 ± 10.7% vs. 3.8 ± 5.7%; p = 0.022; **Figure 3**). Moreover, the rate of applied imaging/mapping techniques was higher in group T: intraoperative DCS (83% vs. 39%; p <0.0001), and preoperative nTMS (94% vs. 15%; p <0.0001)—both linked to the grouping variable TIT.

### Gross Motor Functional and General Clinical Outcome

For the entire group of patients, the postoperative gross motor status was not significantly different from the preoperative condition (full cohort; mean ± SD: 0.8 ± 0.9 vs. 0.7 ± 0.9; p = 0.33). Accordingly, in most patients, gross motor function remained at least unchanged after surgery (n = 54 [89%]; **Table 2**). Twelve (20%) of those patients showed gross motor functional improvement. In contrast, seven patients (11%) presented a deterioration of gross motor function at the time of discharge (**Table 2**); in two of the cases, the cause was not directly related to the removal of tumour tissue in motor eloquent location (case 18: sinus venous thrombosis with fatal outcome; case 34: ischemia inside the posterior limb of the internal capsule, outside the tumour volume, due to vascular damage in the posterior insular region). Of the remaining five cases (8%), four had a pre-existing motor deficit. In all five cases, the degree of deterioration was limited to one scale level.

**Table 2:** Patient outcome.

Extent of resection	
GTR	44 (72%)
RTV (ccm, mean ± SD)	0.8 ± 2.0
RRTV (%, mean ± SD	3% ± 7%
**Gross-motor function and general clinical condition**	
KPS at discharge (median [range])	90 [50;100]
Better	13 (22%)
Same	30 (50%)
Worse [by ≥20 pts]	17 (28%)
KPS after 3 months (median [range])	80 [40;100]
Better	15 (25%)
Same	17 (28%)
Worse [by ≥20 pts]	28 (46%)
Gross motor deficit at discharge	
*Absolute (median grade 0–3 [range])*	1 [0-3]
Any gross motor deficit	31 (51%)
Slight gross motor deficit (MRC 4/5)	20 (33%)
Moderate gross motor deficit (MRC 3/5)	7 (12%)
Severe gross motor deficit (MRC 0–2/5)	4 (7%)
*Relative*	
Better	11 (18%)
Same	43 (71%)
Worse	7 (12%)
* [related to tumour tissue removal]*	*[5 (8%)]*
Due to vascular lesion (distant ischemia)	1 (3%)
Due to sinus thrombosis	1 (3%)
**Survival**	
OS in months (median *[CI]*)	15.0 *[12.0;18.0]*
Death events (percent of total)	59 (97%)
Due to surgery-related complications * [prior to discharge]*	2 (3%) *[1(2%)]*
Due to surgery-unrelated complications	1 (2%)
PFS in months (median *[CI]*)	7.6 *[5.6;9.5]*
Progression or death events (percent of total)	59 (97%)
N patients progression-free alive (rate)	2 (3%)
Death prior to first routine MRI (within 3 months) * [prior to first follow-up]*	5 (8%) *[6 (10%)]*

Outcome data are provided for the full patient cohort (n = 61). CI: 95% confidence interval. SD, standard deviation.

Compared to the preoperative time point, also the general clinical condition of the patients remained statistically unchanged at the time of discharge (both median KPS = 90 [50, 100]; p = 0.6), but deteriorated until the follow-up visit after three months (median KPS = 80 [40, 100]; p = 0.017; **Table 2**). As far as evaluable, none of the patients with postoperative worsening of gross motor function had fully recovered within three months (permanent deficit: n = 4; prior death: n = 1; lost to neurological follow-up: n = 2).

As expected, the degrees of gross motor functional deficit and KPS were negatively correlated, both pre- (r = −0.396; p = 0.002) and postoperatively (r = −0.371; p = 0.003).

Univariate and subsequent multivariate analysis of our data (full cohort) identified the CST-overlap, which was strongly correlated with the (preoperative) degree of gross motor deficit (Spearman’s rho = 0.518; p = 0.00002), as the best predictor of postoperative gross motor function worsening. Accordingly, *post hoc* Wilcoxon tests showed a statistical trend towards a higher CST-overlap in the group with gross motor worsening (W = 196; p = 0.080). Moreover, a significant Spearman correlation was observed between the CST-overlap and the degree of postoperative gross motor deficit (rho = 0.382; p = 0.003).

Despite the significantly more probable tumour infiltration of the CST in group T according to statistical overlap analysis (cf. *Patients*), there was no significant difference between groups (T vs. C) regarding the resection-related change in gross motor function (p = 0.108) or KPS (p = 0.939; **Table 3**). Accordingly, there was no significant difference between groups T vs. C regarding the relative change of the gross motor functional deficit (0.0 ± 0.7 vs. −0.2 ± 0.5; p = 0.192). Notably, cases 18 and 34 were excluded from group comparisons to minimise bias.

**Table 3:** Contingency tables showing group-wise distribution of functional outcome.

		Postoperative change					
		Gross motor function			KPS		
		better	same	worse	better	same	worse
**Group**	**T** (n = 33)	7	21	5	8	17	8
	**C** (n = 26)	5	21	0	5	15	6
**Overall** (n = 59)		12 (20%)	42 (71%)	5 (8%)	13 (22%)	32 (54%)	14 (24%)

The two 3 × 2 contingency tables demonstrate the postoperative change of gross motor function (left) and of the KPS (right) across groups T vs. C. Fisher’s exact test showed no significant group influence on gross motor (p = 0.108) or KPS outcome (p = 0.939). Group T, TIT. Group C, control, no TIT. Cases 18 and 34 (with postoperative deficits unrelated to direct mechanical injury of the CST/M1) were excluded. cf. **Supplementary Table S4** for a complementary statistical analysis in the subgroup of patients with intraoperative neuromonitoring (which largely agreed with the key results presented here).

### Resection Outcome

GTR was achieved in 72% of all cases (**Table 2**, translating to n = 17 subtotal resections overall). Six of the subtotal resection (T group: n = 3; C group: n = 3) were planned as partial resections, due to a clinically critical tumour mass effect combined with the existence of distant/satellite CE-tumour lesions (n = 2) or CE-tumour infiltration of functionally very eloquent midline structures (n = 4). In another three cases of subtotal resection (including one T group patient), parts of M1 were spared, according to intraoperative confirmation by DCS. In the remaining eight cases, other intraoperative findings led to a less rigorous surgical strategy (n = 4), or the cause of incomplete resection remained unclear (n = 4).

According to the high GTR rates, RTV was low (mean: 0.8 ± 2.0 ccm; **Table 2**), corresponding to an average RRTV of 3% ± 7% across all patients (n = 61).

Chi-squared tests to compare GTR between groups T and C showed no significant difference overall (T: 74% vs. C: 69%; p = 0.883) and for the subgroup of n = 55 patients with *a priori* intended GTR (i.e., excluding the six cases with intended incomplete tumour debulking; T: 81% vs. C: 78%; p = 1.000; **Table 4**), thus supporting the hypothesis of non-inferiority of the T group despite the higher average CST-overlap.

**Table 4:** Contingency tables showing group-wise distribution of resection completeness.

		Patient cohort			
		Full (n = 61)		GTR intended (n = 55)	
		GTR	subtotal	GTR	subtotal
**Unmatched groups**	**T**	26	9	26	6
	**C**	18	8	18	5
		Full Match (n = 52)*		Match GTR intended (n = 46)**	
		**GTR**	**subtotal**	**GTR**	**subtotal**
**PSM-paired groups**	**T**	20	6	20	3
	**C**	18	8	18	5

Four-fold contingency tables for the unmatched groups (upper part) failed to show an association of TIT with the extent of resection in the full (unmatched) patient cohort (left; Pearson’s χ² = 0.02; p = 0.883) and in the subset of patients with a-priori intention of GTR (right; Pearson’s n = 55; χ² = 1.2 × 10^−30^, p = 1.000). PSM-paired groups (lower part): the tables demonstrate the distribution of GTR amongst groups matched pairwise using PSM (cf. **Supplementary Tables S1, S2**), pointing towards a higher probability of GTR in the T group for both the full PSM cohort (McNemar’s χ² = 5.04; p = 0.025) and for the subgroup with intended GTR (McNemar’s χ² = 9.33; p = 0.003). Of note, these findings are largely confirmed also for the subgroup of patients with intraoperative neuromonitoring (cf. **Supplementary Table S5** for an overview of the complementary data). *p <0.05; **p <0.01.

To identify and rule out potential confounders, univariate and subsequent multivariate logistic regression analysis and model simplification according to AIC was performed, showing that the best prediction of GTR could be made by the insula-overlap (p = 0.090) with the preoperative tumour volume (p = 0.017). When excluding, however, the six patients with *a priori* intended subtotal resection, the single best predictor was the insula-overlap (p = 0.096). To control for the influence of both parameters, PSM was performed and identified optimally matched pairs between groups T and C based on full patient cohort (i.e., full match, n = 52; **Supplementary Table S1**) and on the reduced dataset of patients with intended GTR (n = 46; **Supplementary Table S2**).

Comparison of GTR across the optimally matched patient pairs showed higher GTR in the T group, for the full match (T: 77% vs. C: 69%; p = 0.025) and for the matched patients with intended GTR (T: 87% vs. C: 78%; p = 0.003; **Table 4**).

### Survival Outcome

**Figure 4:**
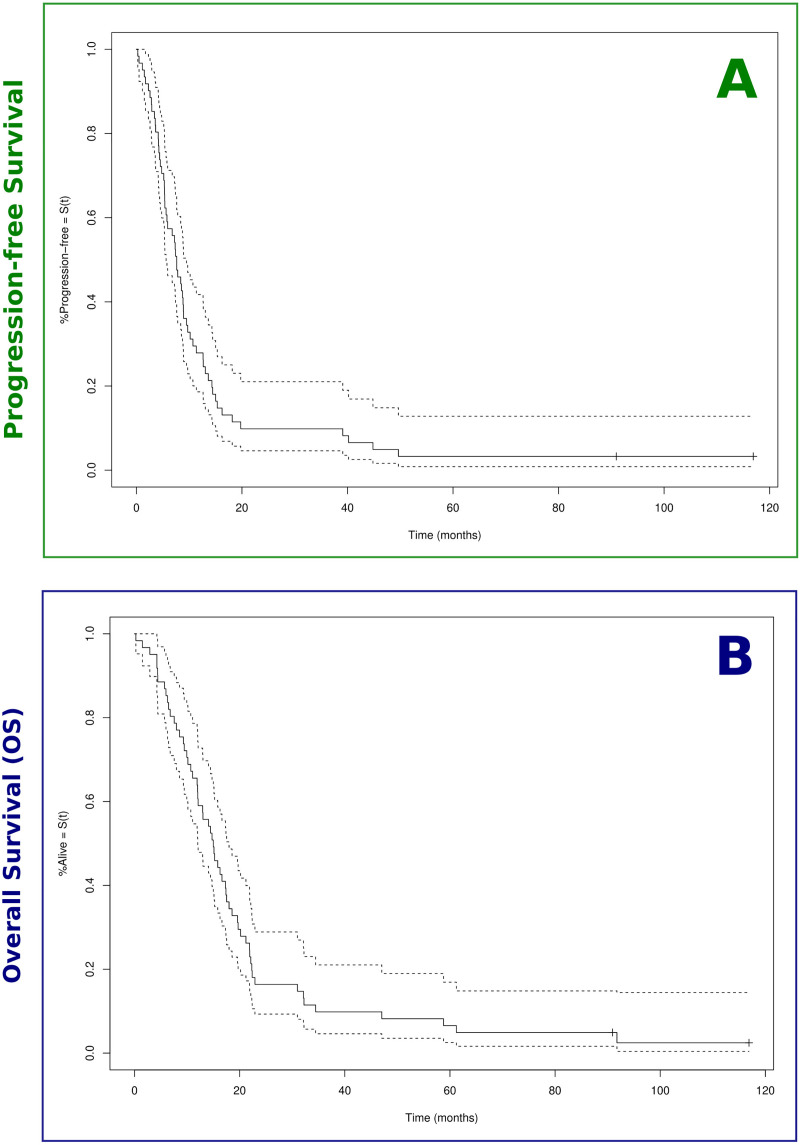
Kaplan–Meier survival curves. Estimated survival proportions according to Kaplan–Meier are displayed for the full patient cohort (n = 61). **(A)** Median progression-free survival (PFS) was 7.6 months. **(B)** Overall survival (OS) was 15.0 months (OS; cf. **Table 2**). The associated lower and upper bounds of the 95% confidence interval are displayed as dashed lines. Censoring events are indicated by vertical ‘ticks’.

The median PFS was 7.6 months (95% confidence interval [CI] = 5.6–9.5 months) and the median OS was 15.0 months (CI = 12.0–18.0 months; **Table 2**; **Figure 4**). Two female patients (ages 32 and 50 years) were still alive, progression-free and in good clinical status at the time of manuscript preparation (i.e., 117 and 91 months postoperatively). Three patients (with the first two affiliated to the T group) suffered from early postoperative death due to therapy complications (case 18: thrombosis of transverse sinus, 8 days OS; case 22: colostasis/ileus with secondary complications during radiochemotherapy, 88 days OS; case 21: subdural hygroma with trapping valve mechanism, complicated re-surgery and subsequent death, OS 129 days). Another patient from group T (case 2) underwent partial tumour resection to achieve mass reduction but remained in too limited general clinical condition (i.e., KPS 60 postoperatively) to receive adjuvant treatment; he died before the first follow-up time point (OS 45 days), probably due to tumour progression. All other patients were alive at the time point of the first follow-up MRI three months postoperatively. Of note, median survival times in the PSM-paired subset of 46 patients with *a priori* intended GTR (cf. *Resection Outcome*) were 8.12 [5.92, 9.72] months (PFS) and 14.9 [12.1, 17.5] months (OS).

We hypothesised that the achievement of tumour mass reduction, positively influenced by the use of TIT, could lead to improved survival. Uni- and multivariate Cox proportional hazards regression analysis followed by standardised model simplification identified GTR as the only variable, which significantly predicted PFS (p = 0.002; **Table 5**). In contrast, the optimal model to predict OS was composed of GTR and age (Wald test p = 0.00007; **Table 5**). Thus, for OS analysis, PSM was performed leading to an optimal pairwise balance of age (median 60 vs. 59 years; p = 0.961) between groups with vs. without GTR (2:1 match; cf. **Supplementary Table S3**), hence allowing largely unconfounded group comparison.

**Table 5:** Cox proportional hazards regression models best describing survival outcomes.

Outcome	Feature	Coefficients			Wald statistic	
		Estimate	Hazard ratio	SE	z value	*p*-value
PFS	GTR	−1.011	0.364	0.308	−3.284	0.001**
OS	Age	0.040	1.041	0.012	3.238	0.001**
	GTR	−1.125	0.325	0.311	−3.612	0.0003***

Results of the winning cox proportional Hazards regression models are shown for PFS (upper line) and OS (lower lines). The respective features were considered for PSM. SE, standard error. **p <0.01; ***p <0.001.

**Figure 5:**
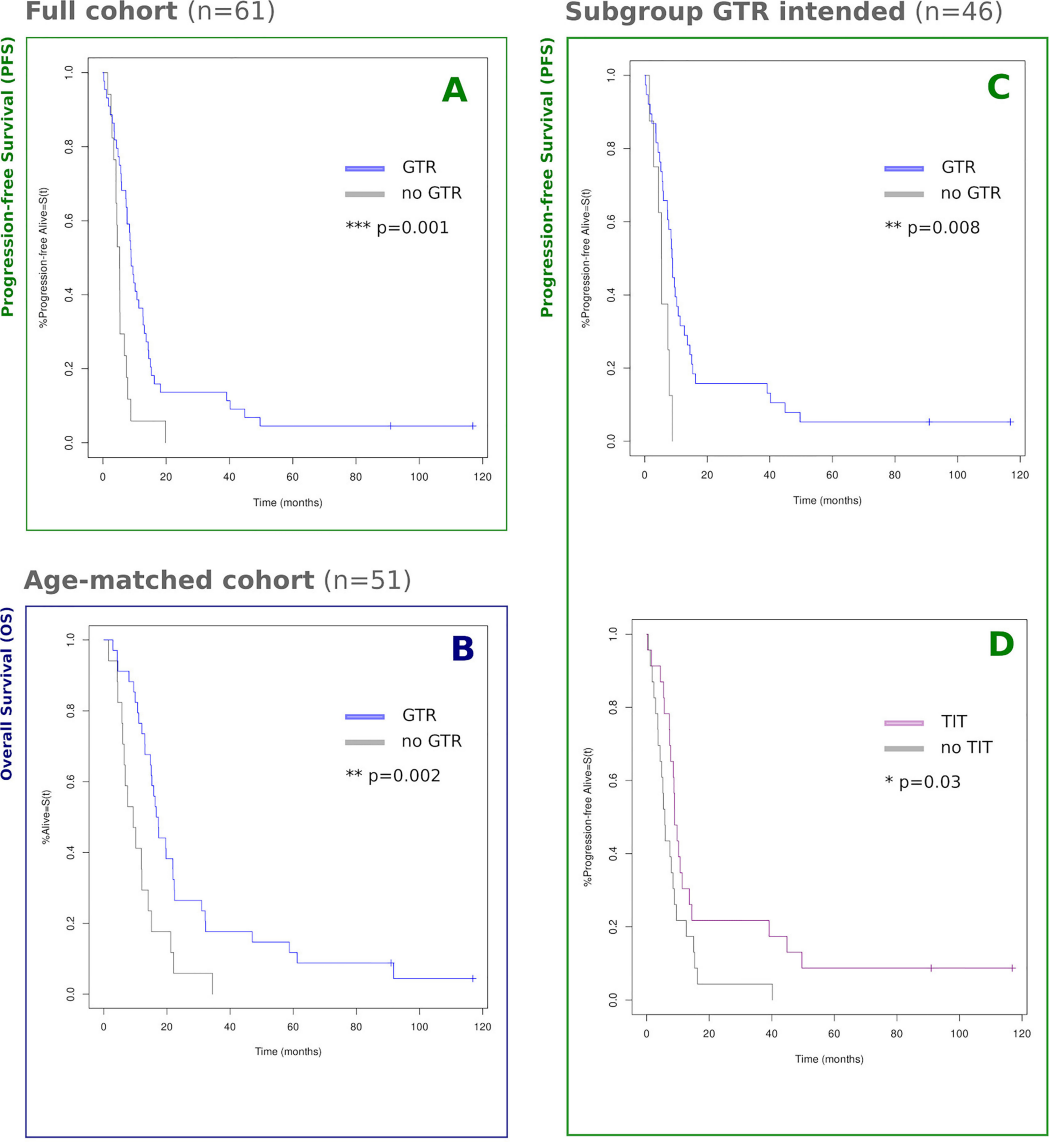
Kaplan–Meier survival curves by group. Estimated survival proportions according to Kaplan–Meier statistics are displayed, grouped by GTR/TIT. Censoring events are indicated by vertical ‘ticks’. **(A)** Full cohort of n = 61, GTR vs. no GTR: 8.9 vs. 5.3 months [χ² = 10.6; p = 0.001]; **(B)** PSM age-paired cohort of n = 51, GTR vs. no GTR: 17.0 vs. 9.3 months [χ² = 9.7; p = 0.002]; **(C, D)** Subgroup of n = 46 patients a-priori eligible for GTR, pairwise balanced for PTV and insula-overlap using PSM. **(C)** GTR vs. no GTR: 8.8 vs 5.5 months [chi2 = 7.1; p = 0.008] ("p = " is missing); **(D)** TIT vs. no TIT: 8.9 vs. 5.8 months [χ² = 4.7; p = 0.030]. For an overview of PSM raw data, cf. **Supplementary Tables S3** **(B)** and **S2 (C**, **D)**. *p <0.05; **p <0.01; ***p <0.001.

The stratified log-rank test showed GTR to be significantly associated with longer PFS (p = 0.0007; data base: full cohort of n = 60) and with longer OS (p = 0.002; data base: age-matched sample of n = 51; **Figures 5A, B**).

Notably, for the subgroup of patients with intended GTR, controlled for the bias of PTV and insula-overlap, the use of TIT was not only associated with slightly higher GTR rates (cf. *Resection Outcome*) but also with prolonged PFS (p = 0.030; dataset: PTV/Insula-matched sample of n = 46 with intended GTR; **Figures 5C, D**).

## Discussion

In a consecutive single-centre cohort of 61 patients first diagnosed with motor eloquent glioblastoma, we tested the hypothesis that at least equally high GTR rates can be achieved for tumours in comparatively close relationship to the CST without causing major functional harm, when using TIT for comprehensive and individualised resection planning. Moreover, we explored the consequences of TIT-aided resection for survival.

Our results showed that, despite a more critical tumour localisation regarding the CST-overlap, higher GTR rates could be achieved using TIT compared to controls in a PSM-paired sample balanced for all significant independent confounders (i.e., PTV and insula-overlap). This achievement in tumour control was at the cost of a nonsignificantly higher proportion of resection-induced worsening of gross-motor function. Even in this specific cohort of patients with glioblastoma in highly motor eloquent localisation, GTR—optimisable by TIT-aided surgery—still represents the strongest predictor of PFS and OS. Also, the application of TIT translated into a significant increase in PFS.

### Maintenance of Gross Motor and General Clinical Functioning

Our data confirm the expectation of a correlation between general clinical functioning (according to KPS) and gross motor function, none of both parameters yielded statistically significant differences between groups (T vs. C). The significant deterioration in KPS three months postoperatively compared to the status at discharge, however, seems primarily attributable to early tumour recurrence and may additionally reflect the clinical correlates of the adverse effects of radiochemotherapy. With overall five cases of gross motor function worsening caused by direct mechanical injury (8% overall, 15% in group T), corresponding to at least three cases of permanent deficits (5% overall, 9% in group T), overall our results conform to previous publications. Other authors reported permanent deficit rates between 6% (30) and 22% (31, 32) in patients with similar characteristics and technical aids [cf. (8)]. A recently published, comparatively large bi-centric dataset including n = 165 patients with motor eloquent gliomas by Rosenstock et al. (33) found permanent deficits in 13% of all cases, closely agreeing with our findings. Although the difference in directly resection-induced functional outcome between groups C and T did not meet the level of statistical significance (p = 0.108), possibly due to the rareness of the events and the limited sample size (cf. *Strengths and Limitations*), all patients with an unfavourable motor outcome were part of the T group. This can be interpreted as a consequence of the closer relationship between tumour and CST in the T group, thus confirming the results of Rosenstock et al. (33) who identified the structural integrity of the CST (expressed by the average fractional anisotropy) and strongly correlated distance between tumour and CST as the co-principal components of a risk stratification model predicting motor functional outcome in glioma surgery. This corresponds to the results of uni- and multivariable regression analyses of our data identifying the CST-overlap as the best predictor of gross motor functional worsening, which was strongly correlated with the preoperative functional deficit. Moreover, the utmost incomplete recovery of all patients with gross motor functional deterioration who could be neurologically re-evaluated three months later (n = 4) mirrors the evidence from stroke research: the functional reorganisation potential after CST lesion, principally mediated by the recruitment of secondary motor network components, seems largely inefficient (3) and depends critically on the degree of structural CST damage (34).

Notably, the valuable technique of intraoperative (semi-) continuous dynamic mapping of the CST (35) based on the principle of intermittent monopolar subcortical stimulation (36) was established for routine use in our centre only after the period of patient allocation to this study. Recent data from Seidel et al. (37) using this innovative technique in n = 182 cases of motor eloquent brain lesions showed permanent surgery-induced motor deficits in 3% overall, and in 1.7% of the patients caused by direct mechanical injury. Despite limited comparability of their dataset with ours (e.g., due to centre effects and heterogeneous tumour entities including only 45% glioblastoma), it can be assumed that the use of (semi-) continuous dynamic mapping might help to further improve the balance between resection completeness and avoidance of functional harm.

### Towards an Optimised Degree of Resection

Intraoperative fluorescence-guidance of resection (usually by 5-ALA), which was applied in all except for two cases in our study, was a milestone in the history of high-grade glioma surgery (38, 39). To date, 5-ALA fluorescence represents a standard intraoperative tool but not a common outcome measure, since objective quantification methods for residual fluorescence have not yet been established. However, the prognostic value of (quantitative) intraoperative metabolic imaging represents an interesting subject to future investigations. Although there is substantial agreement that fluorescence-guidance improves the completeness of resection (and, thus, hampers comparison with historical cohorts before the introduction of 5-ALA), GTR rates reported in the literature vary strongly in the range between 50 and 80%, even in cohorts with routine use of 5-ALA [cf. (40) for review]. Regardless of the eloquent tumour locations, resection outcomes in our study cohort reached 72% overall (74% in the T group) and 80% GTR in the subgroup with *a priori* intended GTR. Other groups applying similar CST visualisation techniques (including tractography) to inform motor eloquent tumour resections reported GTR rates between 50% (31) and 73–74% (33, 41) in mixed cohorts mainly including high-grade glioma patients, which, however, limits direct comparisons with our study results. Comparing GTR rates between groups with vs. without TIT, we found a significant difference (T: 77% vs. C: 69%; p = 0.025) when using PSM to control for the confounding effects of PTV and insula involvement. This effect appears mainly attributable to TIT rather than to nTMS (alone), i.e., according to the results of Raffa et al. (42) who found a significant improvement in GTR with the use of TIT but nonsignificantly by nTMS alone in a case-control study with three diagnostic arms. Although generally supporting the results of other groups who also found higher GTR rates in patient cohorts of DTI-informed surgery, the resection outcome in our control group was still favourable compared to the previously reported control GTR rates, e.g., in the range of 51–53% (41, 43). This might be due to a better control of confounders in this matched control group taken from the same period. For instance, the use of intraoperative neuronavigation, being inevitably linked to the intraoperative use of functional data like TIT, has been increasingly used over the past decades, and can *per se* contribute to improving the extent of resection. In our study, only four patients (of the control group) were operated without intraoperative neuronavigation (two of them with GTR outcome), thus reducing this bias to a minimum.

The still considerable advantage of TIT-informed surgery (planning), regarding resection completeness, could be explained by (i) better patient selection regarding the objective of GTR without causing relevant functional harm. This primary hypothesis is supported by the fact that two surgeries (8%) in group C vs. only one surgery in group T (3%) were switched to subtotal resection based on intraoperative DCS results. Another explanation is (ii) a higher degree of confidence of the neurosurgeon towards the CST localisation due to the intraoperative availability of functional neuronavigation including TIT results, leading to a more aggressive surgical strategy, at least at a supposedly “safe” distance from the expected location of the CST [generally corresponding to a distance of ≥3–5 mm, dependent on the local anatomy/vascularisation (37)]. Whether or not this effect of TIT could be similarly achieved by the routine use of intraoperative (semi-) continuous dynamic mapping remains to be investigated in direct comparison of both methods.

### Survival

With an overall median OS of 15.0 months and the median PFS of 7.6 months overall, our survival results are in the upper field compared to larger-scale studies of surgically treated primary glioblastoma patients with unspecified tumour localisation reporting median PFS in the range of 6–7 months and median OS between 12 and 15 months (44–48). We hypothesise that the motor eloquent location of tumours predisposes patients for comparatively early tumour detection due to the association with motor symptoms and focal seizures (49), mostly still in a stage eligible for GTR. This assumption is supported by the facts that (i) in our dataset, comparatively high GTR rates were achieved (cf. *Discussion*, *Resection Outcome*), and that (ii) OS outcome data in our GTR subgroup (i.e., median 17.0 months) are in close agreement with median OS of patients with completely resected primary glioblastoma of random localisation [e.g., Kreth et al. (GTR: n = 125): 17.1 months (46); Stummer et al. (GTR: n = 137): 17.9 months (38)].

Regarding the effect of the extent of resection on survival, which is the subject of an ongoing debate [cf., e.g., (50)], we identified GTR but not RTV as the strongest influenceable predictor of survival. Similarly, Kreth et al. (46) found GTR but not subtotal resection to be associated with prolonged survival. Hence, also in motor eloquent tumours, GTR represents the key player to improve survival. Local tumour recurrence (facilitated by incomplete resection) likely affects motor function and, thus, the general clinical condition of the patients, and offers limited second-line local therapy options due to the regional eloquence. Our conclusions are supported by recent data of Hendrix et al. (43), who also identified older age and subtotal resection as the primary hazards for overall survival of patients with motor eloquent high-grade gliomas (although in a mixed cohort of WHO grade 3/4 gliomas).

### Strengths and Limitations

As far as we are aware, this is the first study to systematically assess survival outcome in a consecutive patient cohort composed exclusively of primary motor eloquent glioblastoma patients treated by TIT-guided tumour resection, allowing for first-time demonstration of a significant effect of TIT-driven GTR on survival. This could be achieved using an optimal PSM and a rigorous strategy of data-driven statistical analysis (i.e., selecting features for matching based on regression analysis), particularly well-suited for small-sample studies in rare diseases like motor eloquent glioblastoma.

The non-randomised design, however, remains an important limitation of this study and explains the larger proportion of CST-overlap in the T group, as patients with tumours close to the CST were more likely subjected to TIT than patients with comparatively CST-distant tumours. Even despite this confound, as stated above, we still found a longer PFS in patients undergoing TIT. Therefore, our study supports the idea of a prospective randomised controlled trial to prove the benefits of TIT in glioblastoma surgery. Alternatively, a large multicentre study (combined with PSM-based statistical analysis) could serve to confirm the results, offering a better balance between the sample size and the large variety of potential confounding factors.

Since DTI was performed only in the T group, a direct assessment of the distance/overlap between the tumour and the CST by individual tractography was impossible for both groups. To allow, however, for a group-wise comparison, a standard CST template overlaid with normalised CE-T1w tumour volumes was used. Although being justified by the issue of between-group comparability, this approach comes with the limitation of providing a less precise estimate of motor eloquence (tending to its over-estimation) compared with individual DTI and might not always agree with expert assessments of the scans.

TIT in our study was performed according to the current state-of-the-art in clinical routine, i.e., based on standard DTI and deterministic data processing representing rather robust tools for CST visualisation (4). However, the precision of the tractography results might be further improved by the use of more advanced techniques of (i) diffusion MRI acquisition, (ii) postprocessing and (iii) co-registration techniques, for instance. (i) The use of optimised b-values and kurtosis tensor strategies (51, 52), high angular resolution diffusion imaging (HARDI)/q-ball imaging (53, 54), neurite orientation dispersion and density imaging [NODDI (55, 56)] can improve the tractography results especially in regions of fibre crossings, at the cost of acquisition time [cf. (57)]. This drawback, however, could be at least partly alleviated using accelerated acquisition schemes such as compressed sensing (58). (ii) In addition to image acquisition techniques, probabilistic tractography algorithms (59, 60) and (iii) elastic fusion in addition to linear co-registration of diffusion imaging and structural MRI contribute substantially to precision improvement, especially in the context of brain tumours, where tumour-related changes in cellular water content interfere with diffusion metrics (61). Furthermore, echo planar imaging (EPI)-based diffusion and fMRI acquisitions can be tailored to display the same image distortions, thus rendering the nonlinear registration unnecessary and allowing for a precise seed region definition.

Concerning functional localisation, recent advancements in fMRI acquisition and analysis, such as high-resolution fMRI [cf. (62)] and high-field fMRI/mesoscopic brain mapping (63, 64) might qualify fMRI techniques, along with magnetoencephalography [MEG (65, 66)], as an acceptable alternative to nTMS as a functional localised to inform CST-tractography in the future.

Even when achieving optimal precision in the estimation of the CST localisation using non-invasive techniques like TIT, its intraoperative validity is hampered by the nonlinear brain shift induced by skull/dura opening, which increases along with the loss of cerebrospinal fluid and the quantity of tumour tissue removed [cf. (67)]. This fallacious co-registration mismatch concerning intraoperative neuronavigation can be at least partly overcome by intraoperative renewal of the co-registration with intraoperative imaging [cf. (68)], i.e., intraoperative MRI [iMRI (69)], CT [iCT (70, 71)] and 3D ultrasound [iUS (72–74)]. However, intraoperative neuromonitoring techniques remain the gold standard in the resection of motor-eloquent gliomas (75), and their partial use in this study represents another limitation. For instance, intraoperative neuromonitoring by cortex stimulation and (semi-) continuous dynamic mapping in particular (36, 76) provide excellent tools for repeated estimation of the CST distance from the resection cavity, e.g., guided by TIT-integrated neuronavigation. These techniques are especially appropriate if patients are not monitored behaviourally in an awake surgery setting, which allows the additional monitoring of higher-order brain functions such as motor control (16, 77, 78). It is possible that the *intraoperative* effect of TIT is at least partly outperformed by a comprehensive and rigorous intraoperative neuromonitoring.

### Conclusions

This study shows that incorporating TIT into the pre- and intraoperative workflow enhances the completeness of resection in GTR-eligible motor eloquent primary glioblastoma. This achievement is associated with a non-significant increase in directly resection-related motor function deficits, and leads to prolonged PFS—with subtotal resection representing the primary influenceable hazard of PFS and OS in this patient cohort. A controlled prospective trial is needed to ultimately prove the benefit of TIT in glioblastoma surgery.

## Data Availability Statement

The raw data supporting the conclusions of this article will be made available by the authors, without undue reservation.

## Ethics Statement

The studies involving human participants were reviewed and approved by the Ethics Committee of the Medical Faculty of the University of Cologne. The participants receiving nTMS and/or TIT were filed in an institutional registry, and provided their written informed consent.

## Author Contributions

CWL: study design, data acquisition, data analysis, manuscript draft & editing. AMF: study design, data acquisition, manuscript draft & editing. RL: data analysis; manuscript editing. CN: manuscript editing. CS, AMOP, KJL, NJS, GS, and VN: data acquisition, manuscript editing. TB and HN: data analysis, manuscript editing. MH: proofing of data analysis, manuscript editing. MK: data analysis and manuscript editing. CG: study design and manuscript editing. RG: study design, data acquisition and manuscript editing. All authors listed have made a substantial, direct, and intellectual contribution to the work and approved it for publication.

## Funding

CWL and RG received funding from the German Research Foundation (DfG) for the purchase of the nTMS device (INST 1856/50-1) and as support for the Article Processing Charge (491454339). CWL received additional funding from the Faculty of Medicine of the University of Cologne (Koeln Fortune/Gerok 8/2016).

## Conflict of Interest

The authors declare that the research was conducted in the absence of any commercial or financial relationships that could be construed as a potential conflict of interest.

## Publisher’s Note

All claims expressed in this article are solely those of the authors and do not necessarily represent those of their affiliated organizations, or those of the publisher, the editors and the reviewers. Any product that may be evaluated in this article, or claim that may be made by its manufacturer, is not guaranteed or endorsed by the publisher.

## Glossary

**Table:**

AIC	Akaike information criterion
5-ALA	5-aminolevulinic
ANTs	Advanced Normalisation Tools
CE	Contrast-enhanced/-ing
CST	Corticospinal tract
CST-overlap	Proportional overlap volume between tumour and the ipsilateral corticospinal tract (CST) relative to the respective CST volume
DCS	Direct cortical stimulation
DTI	Diffusion tensor imaging
EPI	Echo planar imaging
FLAIR	Fluid-attenuated inversion recovery
GLM	Generalised linear model
GTR	Gross total resection
iCT	Intraoperative CT
IDH	Isocitrate dehydrogenase
iMRI	Intraoperative MRI
Insula-overlap	Proportional overlap volume between tumour and the ipsilateral insular cortex relative to the respective insular cortex volume
iUS	Intraoperative ultrasound
KPS	Karnofsky performance scale
M1	Primary motor cortex
MEG	Magnetoencephalography
MEP	Motor evoked potential
MGMT	O6-Methylguanin-DNS-Methyltransferase
MNI	Montreal Neuroimaging Institute
MRC	(British) Medical Research Council
MRI	Magnetic resonance imaging
nTMS	Navigated transcranial magnetic stimulation
OS	Overall survival
PD	Progressive disease
PFS	Progression-free survival
PSM	Propensity score matching
PTV	Preoperative CE-tumour volume
RMT	Resting motor threshold
ROI	Region of interest
RRTV	Relative residual CE-tumour volume
RTV	Residual CE-tumour volume
SD	Standard deviation
TIT	TMS-informed tractography
T1w	T1-weighted
WHO	World Health Organisation
