# Supplementary material for: Surgery of Motor Eloquent Glioblastoma Guided by TMS-Informed Tractography: Driving Resection Completeness Towards Prolonged Survival
Source: Front Oncol. 2022 May 27;12:874631. doi: 10.3389/fonc.2022.874631 (PMC9186060; doi:10.3389/fonc.2022.874631)
Supplement: Supplementary file 1 [file Table_1.pdf]

Supplementary Table S1: Groups T and C optimally matched regarding PTV and the proportion of insula overlapping with CE-tumour volume using PSM (full match; n=52). Wilcoxon signed rank tests showed no significant difference of means between groups T vs. C for the matching parameters PTV (21.2 vs. 26.4; p=0.832) and insula overlap (2.88e-05 vs. 1.36e-04; p=0.148). GTR and RTV raw outcome data are provided (shaded in grey).

Subclass (pair)	T group						C group					
	ID	PTV	Insula overlap	Distance	GTR	RTV	ID	PTV	Insula overlap	Distance	GTR	RTV
**1**	4	41.0	0	0.489	0	0.4	14	47.3	3.79E-05	0.468	0	1.7
**2**	28	6.7	0	0.617	0	0.3	44	6.3	0	0.618	0	1.6
**3**	51	38.0	1.53E-08	0.500	1	0	45	37.4	5.41E-05	0.507	1	0
**4**	19	20.7	5.46E-07	0.566	1	0	46	21.5	0	0.562	1	0
**5**	2	44.3	0	0.477	0	3.9	47	62.0	6.66E-06	0.411	1	0
**6**	20	23.5	0	0.555	1	0	48	23.0	0	0.557	1	0
**7**	31	5.1	0	0.622	1	0	49	5.7	0	0.620	1	0
**8**	38	12.4	7.02E-04	0.650	1	0	50	43.4	2.54E-03	0.679	1	0
**9**	32	40.4	0	0.491	0	4.6	52	40.0	0	0.493	0	1.4
**10**	37	29.1	0	0.534	1	0	53	29.0	0	0.535	1	0
**11**	23	9.3	0	0.608	1	0	54	12.0	0	0.598	1	0
**12**	36	3.7	1.06E-05	0.628	1	0	16	2.6	0	0.631	1	0
**13**	22	50.7	3.50E-07	0.453	1	0	55	52.9	3.09E-05	0.447	0	0.8
**14**	13	9.1	0	0.608	1	0	56	10.3	9.17E-05	0.611	1	0
**15**	18	51.5	0	0.450	1	0	57	109.7	2.93E-06	0.253	0	1.4
**16**	5	12.5	0	0.596	1	0	58	13.4	3.97E-06	0.593	0	1.9
**17**	10	30.1	0	0.530	1	0	59	30.2	4.30E-06	0.530	0	12.1
**18**	30	1.4	0	0.635	1	0	60	1.8	0	0.634	1	0
**19**	15	18.2	7.31E-06	0.575	1	0	61	17.5	0	0.577	1	0
**20**	8	18.4	0	0.574	0	0.4	17	18.2	0	0.575	1	0
**21**	9	15.9	0	0.583	1	0	21	15.4	0	0.585	1	0
**22**	1	25.9	0	0.546	0	5.8	39	38.1	6.27E-04	0.551	1	0
**23**	12	13.9	0	0.591	1	0	40	13.7	0	0.591	0	0.8
**24**	34	9.3	0	0.607	1	0	41	12.4	0	0.596	1	0
**25**	26	35.6	0	0.509	1	0	42	34.0	0	0.516	1	0
**26**	24	3.9	0	0.627	1	0	43	1.9	0	0.634	1	0
