# Supplementary material for: Surgery of Motor Eloquent Glioblastoma Guided by TMS-Informed Tractography: Driving Resection Completeness Towards Prolonged Survival
Source: Front Oncol. 2022 May 27;12:874631. doi: 10.3389/fonc.2022.874631 (PMC9186060; doi:10.3389/fonc.2022.874631)
Supplement: Supplementary file 2 [file Table_2.pdf]

Supplementary Table S2: Groups T and C optimally matched regarding PTV and the proportion of insula overlapping with CE-tumour volume using PSM; only cases with a-priori intended GTR were considered (match GTR intended; n=46). Wilcoxon signed rank tests showed no significant difference of means between groups T vs. C for the matching parameters PTV (20.4 vs. 22.8 ccm; p=0.393) and insula overlap (5.3e-05 vs. 1.5e-04; p=0.410). GTR and RTV raw outcome data are provided (shaded in grey).

Subclass (pair)	T group						C group					
	ID	PTV	Insula overlap	Distance	GTR	RTV	ID	PTV	Insula overlap	Distance	GTR	RTV
**1**	2	44.3	0	0.494	0	3.9	14	47.3	3.79E-05	0.487	0	1.7
**2**	28	6.7	0	0.610	0	0.3	44	6.3	0	0.611	0	1.6
**3**	51	38.0	1.53E-08	0.514	1	0	45	37.4	5.41E-05	0.519	1	0
**4**	19	20.7	5.46E-07	0.568	1	0	46	21.5	0	0.565	1	0
**5**	18	51.5	0	0.471	1	0	47	62.0	6.66E-06	0.439	1	0
**6**	35	19.1	0	0.573	1	0	48	23.0	0	0.561	1	0
**7**	31	5.1	0	0.615	1	0	49	5.7	0	0.613	1	0
**8**	38	12.4	7.02E-04	0.632	1	0	50	43.4	2.54E-03	0.641	1	0
**9**	32	40.4	0	0.506	0	4.6	52	40.0	0	0.507	0	1.4
**10**	37	29.1	0	0.541	1	0	53	29.0	0	0.542	1	0
**11**	34	9.3	0	0.602	1	0	54	12.0	0	0.594	1	0
**12**	36	3.7	1.06E-05	0.620	1	0	16	2.6	0	0.622	1	0
**13**	23	9.3	0	0.603	1	0	56	10.3	9.17E-05	0.605	1	0
**14**	10	30.1	0	0.539	1	0	59	30.2	4.30E-06	0.538	0	12.1
**15**	7	10.2	4.90E-04	0.627	1	0	60	1.8	0	0.625	1	0
**16**	15	18.2	7.31E-06	0.576	1	0	61	17.5	0	0.577	1	0
**17**	3	18.2	0	0.575	1	0	17	18.2	0	0.575	1	0
**18**	9	15.9	0	0.583	1	0	21	15.4	0	0.584	1	0
**19**	20	23.5	0	0.559	1	0	39	38.1	6.27E-04	0.550	1	0
**20**	12	13.9	0	0.589	1	0	40	13.7	0	0.589	0	0.8
**21**	5	12.5	0	0.593	1	0	41	12.4	0	0.593	1	0
**22**	26	35.6	0	0.521	1	0	42	34.0	0	0.526	1	0
**23**	30	1.4	0	0.626	1	0	43	1.9	0	0.624	1	0
