# Supplementary material for: Surgery of Motor Eloquent Glioblastoma Guided by TMS-Informed Tractography: Driving Resection Completeness Towards Prolonged Survival
Source: Front Oncol. 2022 May 27;12:874631. doi: 10.3389/fonc.2022.874631 (PMC9186060; doi:10.3389/fonc.2022.874631)
Supplement: Supplementary file 3 [file Table_3.pdf]

Supplementary Table S3: Groups with GTR vs. with subtotal resection were optimally age-matched 2:1 PSM (n=51). The goodness of match was confirmed by a paired t-test showing no significant difference of means between groups GTR vs. subtotal resection (mean 61.5 vs. 61.4 yrs; p=0.941). Of note, gender (which made only an insignificant contribution to the Cox Proportional Hazards Regression Model; p=0.06) was also balanced across groups (65% vs. 65% males; p=1); for a better overview, also group affiliation (T vs. C; grey font colour) and survival outcome data are provided (shaded in grey).

Subclass (pair)	GTR group								Subtotal resection group							
	**ID**	**Age**	Gender	TIT (T group)	**Distance**	**Death**	**PFS** (months)	**OS** (months)	**ID**	**Age**	Gender	TIT (T group)	**Distance**	**Death**	**PFS** (months)	**OS** (months)
**1**	12	56	0	1	0.690	1	7.2	16.6	1	41	0	1	0.692	1	2.6	5.7
	43	68	1	0	0.688	1	2.3	21.9								
**2**	36	44	1	1	0.567	1	10.2	31.0	4	47	1	1	0.583	1	6.8	12.0
	47	54	1	0	0.412	1	8.4	13.0								
**3**	15	57	1	1	0.635	1	49.7	61.2	44	57	1	0	0.635	1	2.8	4.3
	9	55	1	1	0.419	1	8.5	15.0								
**4**	53	76	1	0	0.725	1	9.5	15.2	52	76	1	0	0.725	1	5.3	7.5
	21	75	1	0	0.558	1	4.3	4.3								
**5**	38	69	0	1	0.762	1	8.7	14.7	55	69	0	0	0.805	1	5.3	21.2
	54	69	0	0	0.805	1	12.7	19.7								
**6**	30	32	0	1	0.648	0	116.9	116.9	57	59	1	0	0.645	1	19.8	22.1
	49	55	1	0	0.419	1	1.1	16.3								
**7**	60	74	1	0	0.716	1	0.5	4.4	58	72	1	0	0.707	1	4.1	6.0
	41	74	1	0	0.551	1	15.3	17.3								
**8**	10	50	0	1	0.653	0	90.9	90.9	59	87	1	0	0.770	1	7.4	9.3
	61	60	0	0	0.772	1	16.2	22.3								
**9**	19	78	1	1	0.733	1	44.8	47.0	8	51	0	1	0.736	1	5.4	15.1
	3	52	0	1	0.666	1	10.7	21.9								
**10**	45	59	1	0	0.645	1	3.6	8.0	11	59	1	1	0.645	1	4.2	11.9
	16	55	1	0	0.419	1	4.8	15.8								
**11**	35	50	1	1	0.599	1	7.3	10.0	14	51	1	0	0.604	1	7.8	14.1
	56	54	1	0	0.412	1	5.9	17.4								
**12**	13	66	0	1	0.747	1	13.1	19.6	2	65	0	1	0.791	1	1.5	1.5
	39	66	0	0	0.795	1	40.2	58.8								
**13**	23	79	1	1	0.737	1	11.3	13.0	27	79	1	1	0.737	1	3.4	4.4
	34	76	1	1	0.565	1	7.6	12.0								
**14**	51	60	1	1	0.650	1	8.9	11.0	28	59	1	1	0.645	1	5.4	6.5
	7	54	1	1	0.412	1	13.6	22.4								
**15**	6	69	1	1	0.693	1	18.1	32.1	29	42	0	1	0.696	1	4.5	34.4
	26	54	0	1	0.678	1	39.1	91.8								
**16**	25	65	1	1	0.674	1	7.6	9.5	32	67	1	1	0.684	1	4.4	6.8
	5	64	1	1	0.481	1	5.6	10.7								
**17**	22	60	0	1	0.714	1	2.9	2.9	40	62	0	0	0.780	1	8.8	10.1
	42	63	0	0	0.784	1	1.7	32.2								
