# Supplementary material for: Surgery of Motor Eloquent Glioblastoma Guided by TMS-Informed Tractography: Driving Resection Completeness Towards Prolonged Survival
Source: Front Oncol. 2022 May 27;12:874631. doi: 10.3389/fonc.2022.874631 (PMC9186060; doi:10.3389/fonc.2022.874631)
Supplement: Supplementary file 4 [file Table_4.pdf]

Supplementary Table S4: Contingency tables showing group-wise distribution of functional outcome for the subset of patients with intraoperative neuromonitoring. The two 3x2 contingency tables demonstrate the postoperative change of gross motor function (left) as well as of the KPS (right) across groups T vs. C. Fisher's exact test showed no significant group influence on gross motor (p=0.503) or KPS outcome (p=0.696). Group T: TIT. Group C: control, no TIT. Case 18 (with postoperative deficits unrelated to direct mechanical injury of the CST/M1) was excluded.

		**Postoperative change**					
		**Gross motor function**			**KPS**		
		**better**	**same**	**worse**	**better**	**same**	**worse**
**Group**	**T** (n=28)	5	19	4	7	15	6
	**C** (n=10)	1	9	0	1	6	3
**Overall** (n=39)		6 (16%)	28 (74%)	4 (11%)	8 (21%)	21 (55%)	9 (24%)
