# Supplementary material for: Surgery of Motor Eloquent Glioblastoma Guided by TMS-Informed Tractography: Driving Resection Completeness Towards Prolonged Survival
Source: Front Oncol. 2022 May 27;12:874631. doi: 10.3389/fonc.2022.874631 (PMC9186060; doi:10.3389/fonc.2022.874631)
Supplement: Supplementary file 5 [file Table_5.pdf]

Supplementary Table S5: Contingency tables showing group-wise distribution of resection completeness throughout PSM-paired patients with intraoperative neuromonitoring. The four-fold contingency tables demonstrate the distribution of GTR amongst groups matched pairwise using PSM, pointing towards a higher probability of GTR in the T group for both the full PSM cohort (McNemar's $\chi^2=4.0$, $p=0.046$) as well as for the subgroup with intended GTR (McNemar's $\chi^2=6.1$, $p=0.013$). These data based on the subgroup of patients which underwent intraoperative neuromonitoring support the findings of the primary data analysis (full cohort), although the statistical power is limited by the reduced sample size. *$p<0.05$.

		Patient subset with intraoperative neuromonitoring			
		**Full Match (n=20)***		**Match GTR intended (n=18)***	
		**GTR**	**subtotal**	**GTR**	**subtotal**
**PSM-paired groups**	**T**	9	1	9	0
	**C**	8	2	8	1
